# The effects of epidural anaesthesia and analgesia on T lymphocytes differentiation markers and cytokines in patients after gastric cancer resection

**DOI:** 10.1186/s12871-019-0778-7

**Published:** 2019-06-12

**Authors:** Liping Wang, Si Liang, Hong Chen, Yang Xu, Yu Wang

**Affiliations:** 1 Department of Anaesthesiology, Harbin Medical University Cancer Hospital, No. 150 Haping Rd., Nangang District, Harbin, 150081 China; 2 Department of Anaesthesiology, Affiliated Hospital of HeBei University, Baoding, China

**Keywords:** Epidural anaesthesia, General anaesthesia, Patient-controlled analgesia, Immune function, Cluster of differentiation, Cytokines

## Abstract

**Background:**

Epidural use can prevent peri-operative neuro-endocrine stress responses, mitigate pain after surgery, and reduce opioid use, which all lead to immunosuppression.

**Methods:**

Forty patients with gastric cancer were ultimately enrolled into the study. Patients who received general anaesthesia (GA group, *n* = 20) or a combination of general anaesthesia and peri-operative epidural use (EGA group, *n* = 20) were given intravenous analgesia or epidural analgesia, respectively. We collected visual analogue scale (VAS) scores, length of hospital stay, the time of the first passage of flatus and incidence of postoperative nausea and vomiting (PONV). We also collected data on the cluster of differentiation markers (CD)^3+^, CD^4+^, CD^8+^, CD^4+^/CD^8+^, interleukin (IL)-4, IL-6, and interferon (IFN)-γ the day before surgery as well as on postoperative days 1, 3, and 7.

**Results:**

VAS scores and PONV in the GA group were higher than in the EGA group on postoperative day 3. CD^3+^, CD^4+^, and CD^4+^/CD^8+^ T cells declined on postoperative day 3 and nearly recovered to baseline seven days after surgery in both groups. CD^3+^ T cells decreased more in the GA group than in the EGA group. IL-4, IL-6, and IFN-γ increased on postoperative day 3 and nearly recovered to baseline seven days after surgery in both groups. IL-4 and IL-6 increased more in the GA group than in the EGA group. IFN-γ increased more in the EGA group than in the GA group.

**Conclusions:**

A combination of general anaesthesia and peri-operative epidural use can relieve postoperative pain and PONV. A combination of general anaesthesia and peri-operative epidural use decreases immunosuppression in gastric cancer resection.

**Trial registration:**

The study procedures were approved by the Ethics Committee of The Harbin Medical University Cancer Hospital. This study was registered prospectively at http://www.chictr.org.cn/index.aspx on October 10, 2017 (Registered ChiCTR-INR-17012939).

## Background

The stress response induced by surgery can activate the immune regulation mechanism during a systemic inflammatory reaction [1–4]. There are many important contributors to the tumor microenvironment, such as cytokines, chemokines, inflammatory mediators, which exists in many stages of progression to metastasis [5–7]. Some clinical factors, such as general anaesthetics, postoperative pain, and opioid analgesia, have been recognized as immunosuppressive and have influenced the development of tumours [8–14]. In some studies, epidural anaesthesia (EA) was associated with improved overall survival in patients with gastric cancer; in other studies, EA did not improve overall survival [15–17]. EA may reduce cytokines and neuro-endocrine stress immune stimulation, prevent nerve impulses, decrease excitability of the sympathetic adrenal medulla axis, reduce cortisol production, and improve the function of T lymphocytes [18, 19].

Among cytokines, interleukin (IL)-4, IL-6, and interferon (IFN)-γ play prominent roles in chronic inflammation, autoimmunity, infectious diseases and cancer, where they often act as diagnostic or prognostic indicators of disease activity and response to therapy [20, 21]. Therefore, we designed this study to explore the interaction between epidural use and the aforementioned cytokines in patients with gastric cancer.

## Methods

### Patient identification and exclusion

This study was a single-centre, randomized, observer-blinded study. The study procedures were approved by the Ethics Committee of The Harbin Medical University Cancer Hospital. This study was registered prospectively at http://www.chictr.org.cn/index.aspx on October 10, 2017 (Registered ChiCTR-INR-17012939). After the written consent was obtained from study participants, 50 American Society of Anesthesiologists (ASA) I-II patients aged 20–80 yr. who underwent radical resection of gastric cancer in our hospital were enrolled in this study between November 12, 2017, and December 15, 2017. Exclusion criteria were: (1) emergency operations, (2) laparoscopic procedures,(3) neoadjuvant treatment,(4) abnormally white blood cell count, (5) peri-operative transfusion, (6) severe heart, lung, liver, kidney, or endocrine diseases, After confirming understanding of the recruiter’s description of the trial, all the patients signed informed consent forms. Anaesthesia and analgesia protocols were standardized as follows. Patients were randomly assigned to the general anaesthesia (GA) group or epidural anaesthesia (EGA) group by computer-generated codes on the beginning of the study.

### Sample size calculation and masking method

The clear primary outcome of this study was inflammatory response, which was evaluated via the concentration of IL-6. Previously published studies on IL-6 suggested that its standard deviation (SD) in vivo is in the order of 7.6 pg/ml**.** Twenty patients were calculated based on detecting a reduction of 6.8 pg/ml deviation on α-value 0.05 and a power of 0.8. To compensate for potential dropouts, we enrolled Twenty-five patients.

The patients were randomly divided into two groups by computer-generated codes on the time of the study onset. Physicians who completed postoperative assessment and biological detection were blinded to the group assignments throughout the study period until follow-up was completed for final analysis.

### Anaesthesia technique and grouping method

On arrival to the operating room, patients were monitored via electrocardiogram and blood pressure as well as pulse oximetry. Patients randomly assigned to the GA group underwent induction of balanced GA with 0.05 mg/kg midazolam (Enhua Pharmaceutical Co., Jiangsu, China), 0.2 μg/kg sufentanil (Renfu Pharmaceutical Co., Beijing, China), 1–1.5 mg/kg propofol (AstraZeneca Pharmaceutical Co., Shanghai, China), and 0.15 mg/kg cisatracurium (Hengrui Medicine Co., Jiangsu, China). Anaesthesia was maintained with propofol when the bispectral index (BIS) was 40–60, and intraoperative analgesia consisted of remifentanil (Enhua Pharmaceutical Co., Jiangsu, China). Oesophageal temperature was monitored and maintained above 36 °C. Patient-controlled intravenous analgesia (PCIA) with sufentanil (0.5 μg/ml) was available for 72–120 h. The PCIA protocol of the sufentanil group (S group) consisted of 0.5 μg/ml sufentanil (total 300 ml) with a background infusion rate of 4 ml h^− 1^, a bolus dose of 3 ml, and a lockout time of 15 min. Patients in the EGA group were given T_8–10_ epidural anaesthesia before general anaesthesia. An infusion of 0.5% ropivacaine (AstraZeneca Pharmaceutical Co., Shanghai, China) was administered during surgery, and the loading dose (0.5% ropivacaine, 5-7 ml) depended on the height and weight of the patient. GA was induced with 0.05 mg/kg midazolam, 0.2 μg/kg sufentanil, 1–1.5 mg/kg propofol, and 0.15 mg/kg cisatracurium, and anaesthesia was maintained with propofol when the bispectral index (BIS) was 40–60. Patient-controlled epidural analgesia (PCEA) with a combination of 0.2% ropivacaine and 0.5 μg/ml sufentanil was available for 72–120 h. Acute rescue analgesic medications could be used when the VAS score was more than 4 and a bolus of 100 mg tramadol, administered via intravenous injection, was required more than 3 times.

### Indicator and data

A physician who was blinded to the group assignment assessed postoperative pain intensity using the visual analogue scale (VAS) on postoperative days 1, 2, and 3. The demographic data, cancer stage, degree of differentiation, duration of the operation, length of hospital stay, time of the first passage of flatus and incidence of postoperative nausea and vomiting (PONV) were recorded. A physician who was blinded to the group assignment collected data on cluster of differentiation markers (CD)^3+^, CD^4+^, CD^8+^, and CD^4+^/CD^8+^, Interleukin (IL)-4, IL-6, and Interferon (IFN)-γ through peripheral blood (10 ml) on the day before surgery, day (d) 0, and on postoperative days 1, 3, and 7.CD^3+^, CD^4+^, CD^8+^ were measured by flow cytometric analysis. The plasma level of IL-4, IL-6, and Interferon IFN-γ were measured by enzyme-linked immunosorbent assay (ELISA). All samples were measured using three independent experiments.

### Statistical approach

Statistical analysis was performed using SPSS version 22.0 for Windows (IBM Corp., USA). Normally distributed data were expressed as the means ± SDs. Categorical variables were described using frequencies and were analysed using the χ^2^ test. Fisher’s exact test was used for small sample sizes (expected frequencies < 5). We checked for normality of the data with the Shapiro Wilk test and used a one-way ANOVA between the two groups. The results with *P* < 0.05 were considered statistically significant.

## Results

### Patient characteristics

**Fig. 1:**
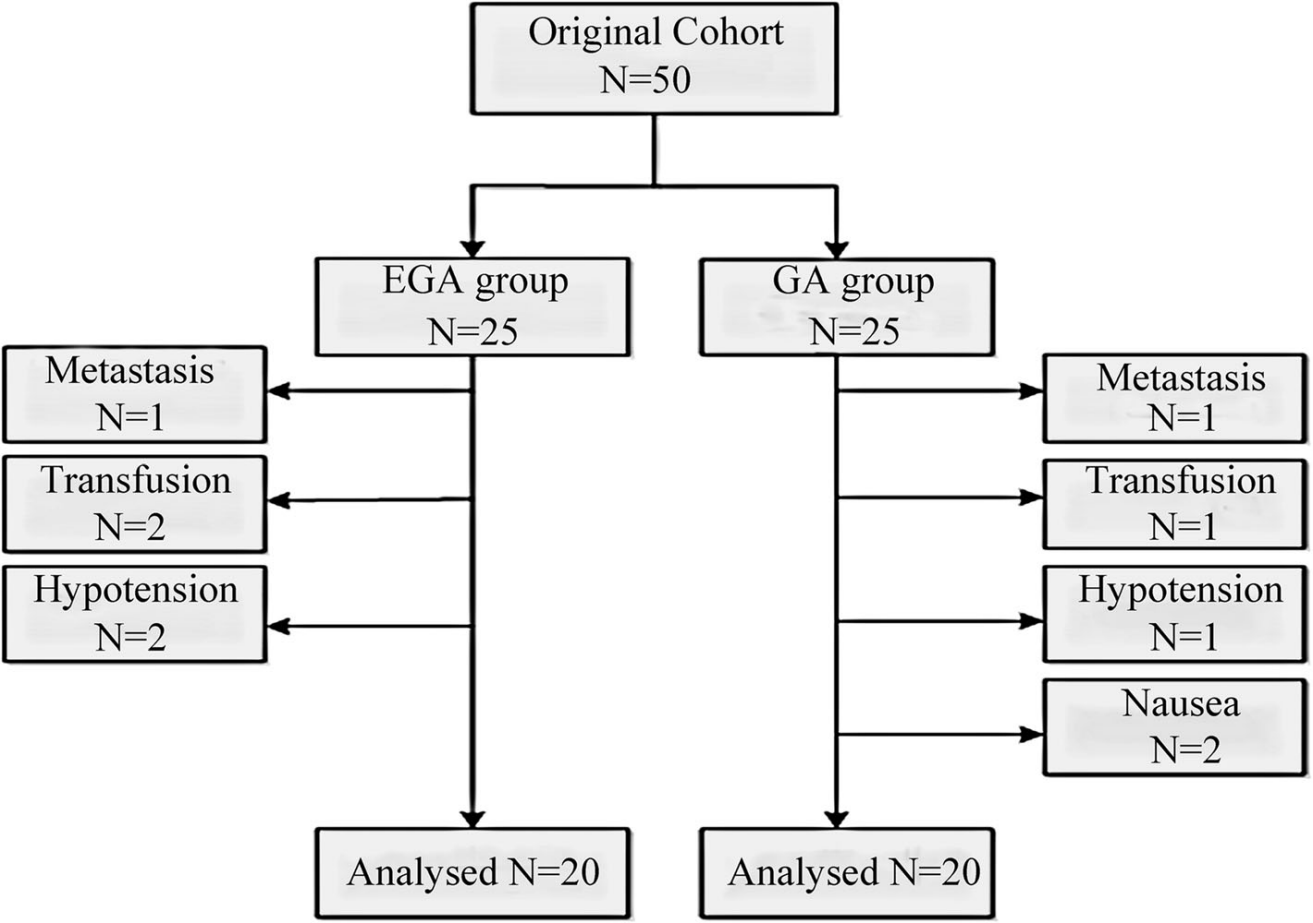
Patient identification and exclusion

Between November 12, 2017, and December 15, 2017, fifty patients were screened for this study, and forty patients were ultimately included in this study. Five patients with metastases or peri-operative transfusions were excluded. Five patients had their analgesic regimen modified because of hypotension and severe nausea (Fig. 1). The two groups were similar with respect to age, height, weight, gender, ASA grade, cancer stage and degree of differentiation, duration of surgery, surgical procedure and surgical manner between the two groups (Table 1).

**Table 1:** Baseline and surgical characteristics of general anesthesia group and epidural combined general anesthesia group

Characteristics	GA group (*n* = 20)	EGA group (n = 20)	*P* value
Age (year)	59.3 ± 6.8	59.7 ± 8.5	0.870
Height (cm)	164.0 ± 6.1	164.5 ± 8.7	0.832
Weight (kg)	66.5 ± 8.4	64.3 ± 7.7	0.394
Gender (male)	11 (55%)	10 (50%)	0.752
ASA grade			
I	2 (10%)	2 (10%)	1.000
II	16 (80%)	17 (85%)	
III	2 (10%)	1 (5%)	
Cancer stage			
I	3 (15%)	4 (20%)	0.925
II	2 (10%)	2 (10%)	
III	12 (60%)	10 (50%)	
Cancer stageIV	3 (15%)	4 (20%)	
Degree of differentiation			
1	7 (35%)	5 (25%)	0.915
2	10 (50%)	11 (55%)	
3	2 (10%)	2 (10%)	
4	1 (5%)	2 (10%)	
Duration of surgery (h)	2.25 (2.00,3.75)	2.25 (2.00,3.50)	0.577
Surgical procedure			
open	20 (100%)	20 (100%)	1.000
minimal invasive	0 (0%)	0 (0%)	
Surgical manner			
total	20 (100%)	20 (100%)	1.000
partial	0 (0%)	0 (0%)	

GA = general anesthesia group

EGA = epidural anesthesia combined with general anesthesia group

ASA = American Society of Anesthesiologists

Cancer stages: I grade-T1, N0, M0/T2, N0, M0/T1, N1, M0; II grade-T3, N0, M0/T4a, N1, M0/T3, N1, M0/T2, N2, M0/T1, N3, M0; III grade-T2, N3, M0/T3, N2, M0/T3, N3, M0/T4a, N2, M0/T4a, N3, M0/ any T4b, any N, M0; IV grade, any T, any N, M1

Degrees of differentiation: Degree1, poorly differentiated; Degree2, moderately differentiated; Degree3, well differentiated; Degree 4, other/unknown differentiated

### Association between epidural use and postoperative variables

**Fig. 2:**
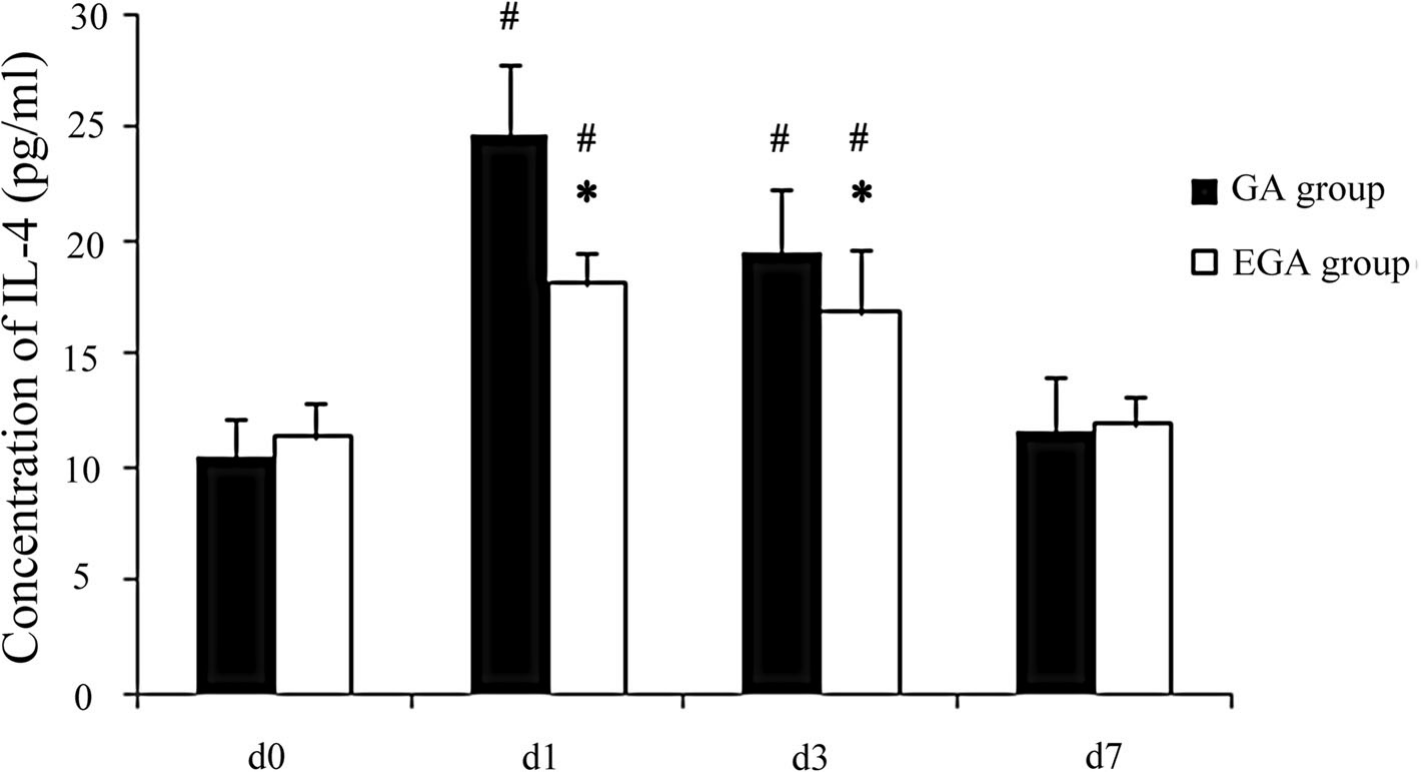
Comparison of IL-4 between the general anaesthesia group and epidural combined with general anaesthesia group on day (d) 0, the day before surgery, and on postoperative days 1, 3, and 7

**Fig. 3:**
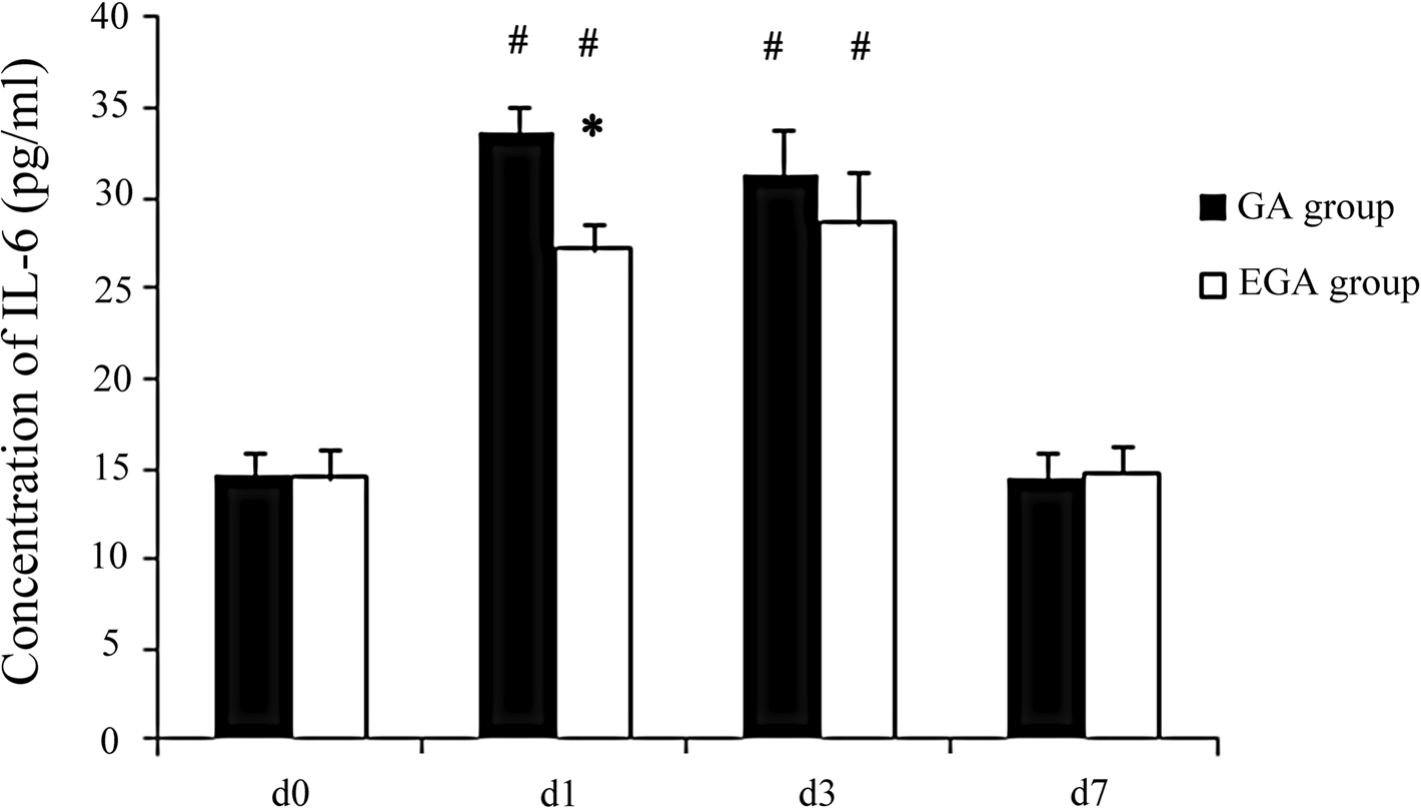
Comparison of IL-6 between the general anaesthesia group and epidural combined with general anaesthesia group on day (d) 0, the day before surgery, and on postoperative days 1, 3, and 7

**Fig. 4:**
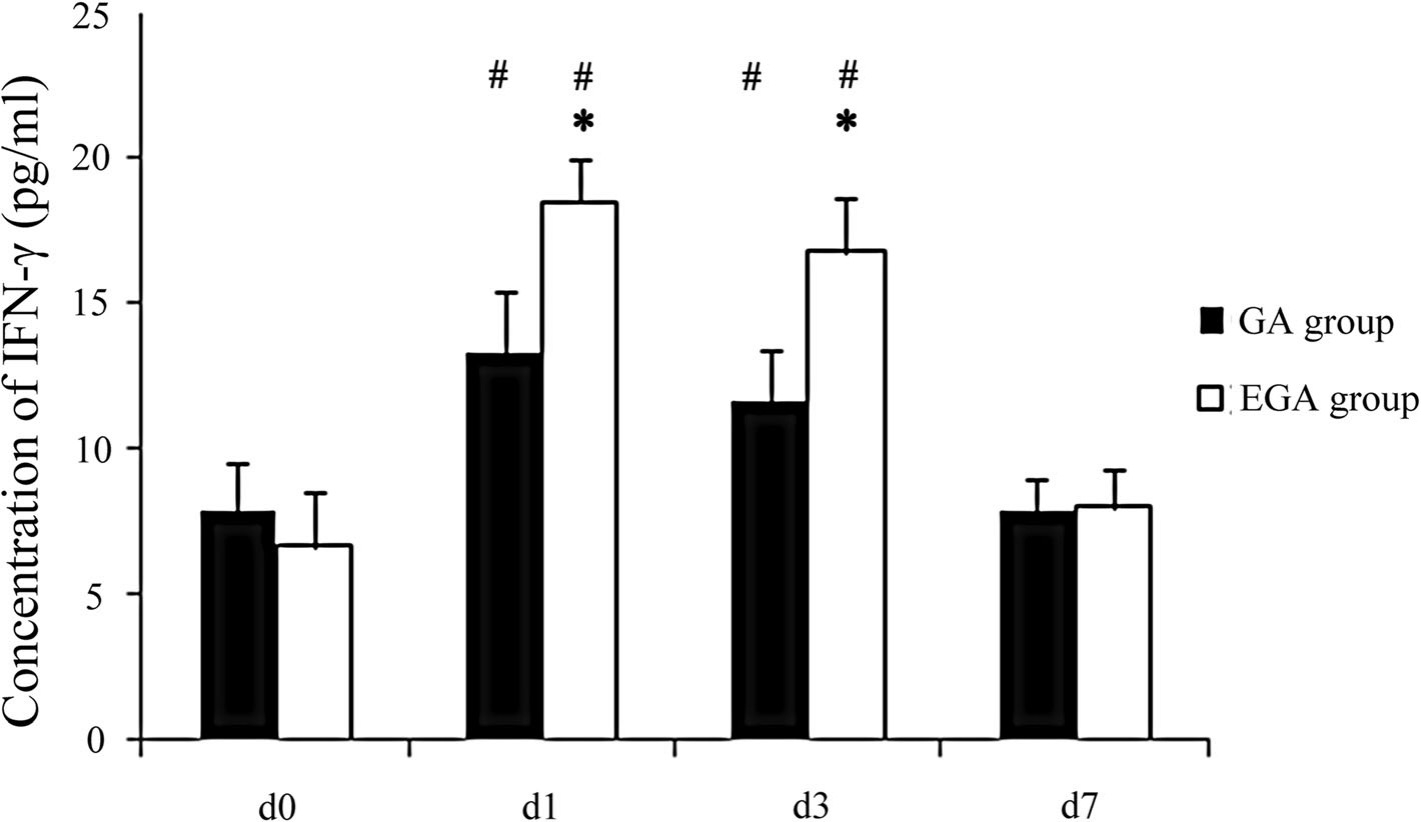
Comparison of IFN-γ between the general anaesthesia group and epidural combined with general anaesthesia group on day (d) 0, the day before surgery, and postoperative days 1, 3, and 7

The VAS scores in the GA group were higher than in the EGA group on postoperative day 3, and the incidence of PONV in the EGA group was lower (*P* < 0.05, Table 2). There were no differences in days of analgesia, the time of the first passage of flatus, and length of hospital stay. CD^3+^, CD^4+^, and CD^4+^/CD^8+^ T cells declined on postoperative day 3 (*P* < 0.05) and nearly recovered to baseline seven days after surgery in both groups. CD^3+^ T cells decreased more in the GA group than in the EGA group (*P* < 0.05, Table 3). IL-4, IL-6, and IFN-γ increased on postoperative day 3 (*P* < 0.05; Figs. 2, 3, and 4) and nearly recovered to baseline seven days after surgery in both groups. IL-4 and IL-6 increased more in the GA group than in the EGA group (*P* < 0.05, Figs. 2 and 3). IFN-γ increased more in the EGA group than in the GA group (*P* < 0.05, Fig. 4).

**Table 2:** The VAS scores of patients in the postoperative days 1, 2, 3 between general anesthesia group and epidural combined general anesthesia group

Characteristics	GA group (n = 20)	EGA group (n = 20)	*P* value
VAS scores			
POD1	3 (2,5)	2 (1,4)	0.004*
POD2	2 (1,4)	1 (0,3)	0.003*
POD3	1 (0,3)	0 (0,2)	0.003*
Days of analgesia	3 (3,5)	3 (3,5)	0.527
Days of flatus time	3 (3,4)	3 (2,4)	0.764
Nausea and vomiting (%)	8(40%)	1 (5%)	0.020*
Length of stay	11 (9,12)	11 (9,12)	0.795

VAS=Visual analogue scale (VAS) scores

POD = Postoperative day

*: Compare between two groups, *P* < 0.05

**Table 3:** Comparison of T lymphocyte subsets of patients in general anesthesia group and epidural combined general anesthesia group

Characteristics	Group	d0	d1	d3	d7
CD3^+^ (%)	GA	54.3 ± 3.5	42.5 ± 2.3#	44.6 ± 3.2#	51.8 ± 6.7
	EGA	55.6 ± 4.1	46.1 ± 2.9#*	48.1 ± 2.2#*	51.2 ± 6.9
CD4^+^ (%)	GA	32.4 ± 4.3	21.5 ± 3.4#	23.2 ± 1.6#	31.7 ± 2.8
	EGA	34.5 ± 3.5	23.4 ± 3.5#	24.5 ± 2.1#	33.0 ± 2.0
CD8^+^ (%)	GA	21.1 ± 1.8	20.7 ± 1.8	21.0 ± 1.9	20.9 ± 1.3
	EGA	21.5 ± 1.2	21.2 ± 1.1	22.9 ± 2.0	21.3 ± 1.5
CD4^+^/CD8^+^	GA	1.5 ± 0.5	1.0 ± 0.2#	1.0 ± 0.1#	1.5 ± 0.2
	EGA	1.6 ± 0.5	1.2 ± 0.2#	1.2 ± 0.3#	1.5 ± 0.1

CD = Clusters of Differentiation

#: Compare to d0 *P* < 0.05, *:Compare between two groups, *P* < 0.05

## Discussion

In our study, we found a beneficial effect of epidural use. The VAS scores in the GA group were higher than those in the EGA group on postoperative day three. The incidence of PONV was higher in the GA group. It has been proven that epidural anaesthesia can provide better analgesia after gastric cancer surgery, which was consistent with our study [22]. Epidural administration of anaesthesia and analgesia is considered a technique with risk of complications, such as neuraxial haematoma, hypotension, pruritus; the subjective experience of anaesthetists often leads to the failure of epidural anaesthesia and analgesia [23, 24]. In our study, there were two patients in the EGA group and one patient in the GA group who could not use EA because of hypotension.

Many studies have reported that opioids impair the peri-operative immune system and increase vascular permeability, and the use of epidural anaesthesia may prevent these peri-operative immunosuppressive changes during major surgery because it can decrease neuro-endocrine stress responses [18, 25–28]. Immune surveillance is the primary indicator for stopping the metastasis of tumours, and immunosuppression may destroy the defensive barrier [29]. Clinical events that may lead to immunosuppressive changes facilitated by surgery include injury, pain, and use of anaesthetic medications [30–32]. CD^3+^ T cells, CD^4+^ T cells, and CD^8+^ T cells are the main cells which take part in antitumour immunity [33]. The ratio of CD^4+^/CD^8+^ cells decreases as the serum cortisol levels increase [34, 35]. In our study, CD^3+^, CD^4+^, and CD^4+^/CD^8+^ T cells were inhibited and had a negative trend. All patients’ CD^3+^ and CD^4+^ T cells decreased to different degrees on the first postoperative day, but in the GA group, patients’ CD^3+^ T cells significantly decreased and recovered to preoperative levels until the seventh postoperative day. The change in CD^8+^ T cells was not statistically significant. These results are consistent with some prior studies [36, 37],they described a lymphocyte depressing factor that was present in the serum of patients in the GA group but was absent in the serum of patients in the EGA group.

Many cytokines can modulate the immune system. Pro-inflammatory cytokines may favour tumour progression. Activated biological cascades lead to immunosuppression, which affects the immune response and decreases IL-4 and IFN-γ [38, 39]. As a pro-inflammatory factor, IL-4 stimulates the proliferation of B cells and participates in the differentiation of Th2 cells. The combination of the cytokine IL-6 with prostaglandin 2 can reduce the production of the immune factor IL-2 by Th1 cells and affect the activation of NK cells [40]. IL-6 has a major influence on the proliferation, survival and metastatic properties of cancerous cells, and studies using preclinical mouse models indicate that treatments targeting IL-6 or its receptor display therapeutic efficacy as anticancer agents [20].In our study, the pro-inflammatory cytokines IL-4 and IL-6 increased more in the GA group than in the EGA group, which may indicate that immune function was less suppressed in the EGA group. IFN-γ can kill tumour cells directly and transform Th0 cells into Th1 cells, which play a large role in the activation of NK cells and T cells. The level of IFN-γ expression embodies the memory ability of this antitumour cytokine [41, 42]. In our study, IFN-γ increased more in the EGA group than in the GA group, which may indicate that immune function was less suppressed in the EGA group. Therefore, in our study, EA was able to relieve postoperative pain and PONV, was able to decrease immunosuppression, and may have reduced inflammatory-associated metastasis in gastric cancer.

There were a few limitations in our study. First, we only measured some T cell subsets and some important immune factors. The immune system is a complex system. There are many immune cells, such as NK cells, and other immune factors, such as IL-2 and IL-12, which may be investigated in the future because these antitumourigenic cytokines were increased by EA. Second, these patients’ long-term outcomes, such as long-term survival, recurrence rates and metastasis rates, could not be obtained at present. We will evaluate these results in 3 to 5 years.

## Conclusions

A combination of general anaesthesia and peri-operative epidural use can relieve postoperative pain and PONV. A combination of general anaesthesia and peri-operative epidural use decreases immunosuppression in gastric cancer resection.

## Data Availability

All data generated or analysed during this study are included in this published article, and can be freely available to any scientists wishing to use. The data supporting our findings can be found in http://www.chictr.org.cn/index.aspx (Registered ChiCTR-INR-17012939), but identifying patient data would not be shared. All the authors agreed to share their data.

## Abbreviations

(NK) cell: Natural killer cell
ASA: American Society of Anesthesiologists
BIS: Bispectral index
EGA: Epidural general anaesthesia
GA: General anaesthesia
IFN: Interferon
IL: Interleukin
PCEA: Patient-controlled epidural analgesia
PCIA: Patient-controlled intravenous analgesia
PONV: Postoperative nausea and vomiting
VAS: Visual analogue scale
